# Acute Colonic Pseudo-Obstruction in an Elderly Female Patient With Chronic Constipation

**DOI:** 10.7759/cureus.44789

**Published:** 2023-09-06

**Authors:** Riddhi R Machchhar, Nusha Fareen, Viraj Shah, Jay Vida

**Affiliations:** 1 Internal Medicine, Rowan University School of Osteopathic Medicine, Stratford, USA; 2 Internal Medicine, Hackensack Meridian Health Ocean University Medical Center, Brick, USA; 3 Internal Medicine, Rajarshee Chhatrapati Shahu Maharaj Government Medical College, Kolhapur, IND

**Keywords:** general gastroenterology, internal med, geriatric patient, multiple comorbidities, chronic constipation, acpo, ogilvie's syndrome

## Abstract

Acute colonic pseudo-obstruction (ACPO), or Ogilvie’s syndrome, is an acute colonic dilatation without mechanical obstruction; it is most commonly seen in severely ill or postoperative patients. While this syndrome has no clear pathophysiology, it is diagnosed when the cecum and right colon expand without physical obstruction. This condition can lead to perforation and intestinal ischemia. Ogilvie’s syndrome is associated with a relatively high morbidity and mortality rate. The diagnosis of ACPO can be often missed due to its vague symptoms such as bloating, abdominal distention, abdominal pain, nausea and vomiting, and severe constipation. We report the case of an 82-year-old female patient who had a unique diagnosis of ACPO, or Ogilvie’s syndrome, overshadowed by the diagnosis of severe constipation. This case highlights the importance of maintaining a high index of suspicion and early diagnosis of symptoms that can rapidly become dangerous.

## Introduction

Acute colonic pseudo-obstruction (ACPO) or Ogilvie's syndrome is a condition that can occur due to many underlying health conditions, surgery, infection, electrolyte imbalances, or the use of medications like narcotics or anticholinergics [1]. It is seen more commonly in older adults aged over 60 years and is twice as common in men compared to women [2]. The incidence of ACPO is approximately 100 cases per 100,000 hospital admissions annually [2]. ACPO occurs due to an imbalance of the colonic motor system. Stimulatory neurotransmitters like acetylcholine are produced less frequently than inhibitory neurotransmitters like nitrous oxide and vasoactive intestinal peptides [2]. The onset of ACPO may be gradual (over three to seven days) or rapid (within 24-48 hours) [3].

Factors such as acute or chronic illnesses, childbirth, trauma, surgery, and drugs inhibiting parasympathetic outflow can lead to ACPO [4,5]. Postoperative ACPO typically presents three to five days following surgery [6]. In Ogilvie's syndrome, mortality is very high if bowel ischemia or perforation develops (40%) compared to the rate of 15% without ischemia and perforation [7]. Symptoms of this condition are of wide range and varying, with the most common complaints being abdominal distension and pain (80%), nausea and vomiting (60%), and obstipation (60%) [1]. Appropriate diagnosis is possible with the right clinical and radiological profile. Conservative treatment is successful in 83-96% of patients without peritoneal signs or evidence of perforation within two to six days. These therapies may be continued for up to 48-72 hours, with a plan to shift the patient to a different intervention if an improvement is not seen [8]. More intense and aggressive treatments include neostigmine, decompression done by colonoscopy, or a cecostomy or colectomy [1, 8]. We present the case of an 82-year-old female with multiple comorbid conditions and a history of severe constipation that complicated the diagnosis and treatment of her Ogilvie's syndrome.

## Case presentation

An 82-year-old female presented to the emergency department (ED) with a complaint of abdominal pain. She had been experiencing generalized abdominal pain and soreness, which she attributed to constipation. The patient had not had a bowel movement for three days before visiting the ED, despite using multiple bisacodyl suppositories. Her past medical history included chronic obstructive pulmonary disease (COPD), congestive heart failure (CHF), paroxysmal supraventricular tachycardia (SVT), peripheral artery disease (PAD), hypothyroidism, chronic constipation, and uterine cancer status-post hysterectomy. Two weeks before her presentation, the patient had been hospitalized for three days to address a COPD exacerbation and had been discharged home with a prednisone taper. During the interview, the patient reported that she felt abdominal discomfort at rest, as well as bloating, dry mouth, generalized weakness, and a headache. She denied other pertinent systemic symptoms. A physical exam revealed a flat abdomen, soft, with mild tenderness to palpation. The patient reported experiencing right upper quadrant (RUQ) pain and some distension. Auscultation of bowel sounds was hypoactive. No rebound or muscle guarding was present, and no hepatosplenomegaly or pulsating abdominal mass was appreciated.

**Figure 1:**
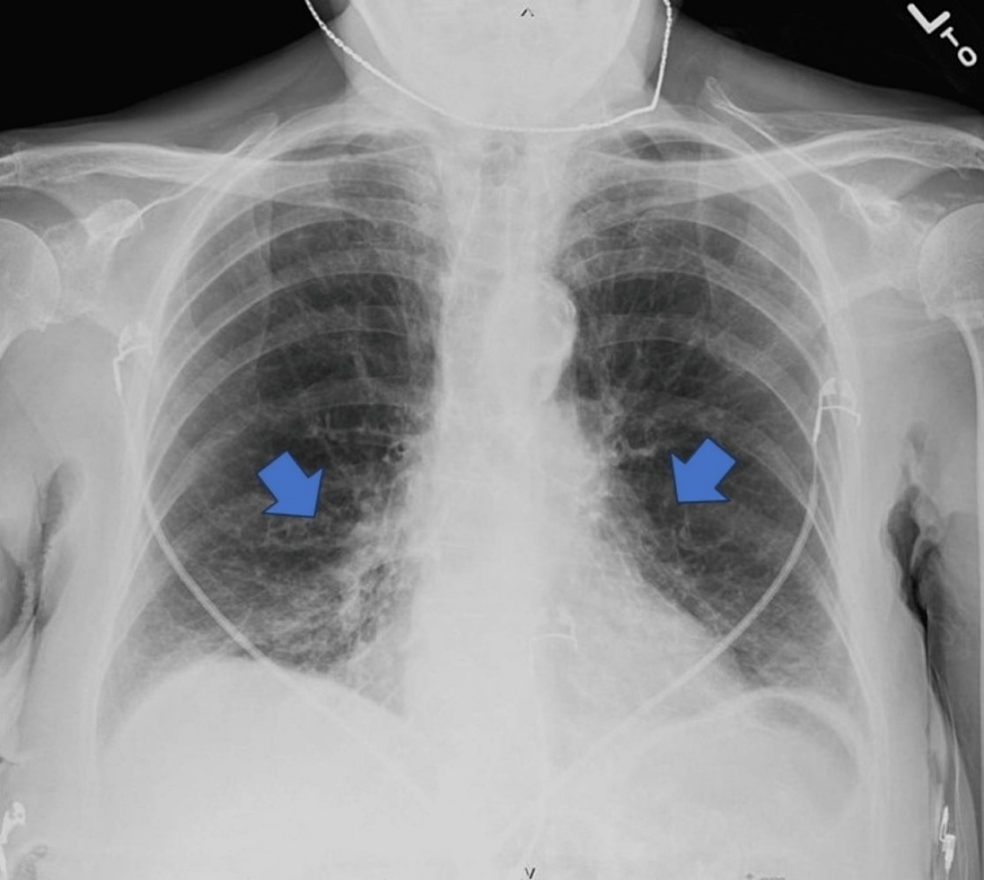
Portable chest radiograph Lungs: well-inflated. Mild chronic interstitial disease with emphysematous changes, stable. Faint mild bibasilar infiltrates. No pneumothorax

Upon admission, the patient's white blood cell count was elevated at 16.6 x 103/uL (reference range: 4.5-11), and her thyroid stimulating hormone was low at 0.115 uIU/mL (reference range: 0.300-4.500). A chest X-ray revealed mild chronic interstitial disease with emphysematous changes consistent with her COPD and mild bibasilar infiltrates (Figure 1).

**Figure 2:**
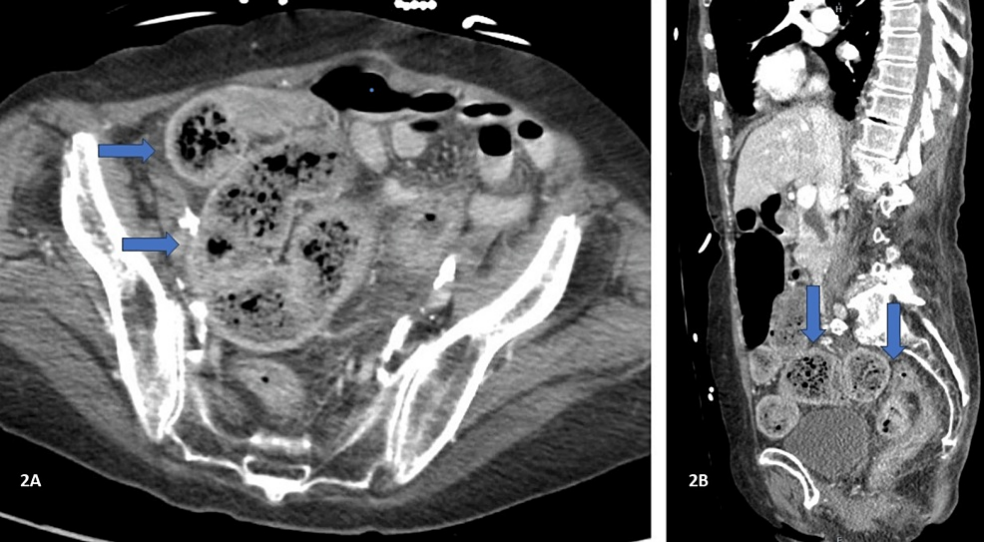
CT with contrast of the abdomen and pelvis - image 1 Impression: a large amount of stool within the colon, which is compatible with constipation. There is colonic thickening and induration involving the rectum and sigmoid colon; there is marked dilatation of the cecum. Compatible with colitis in the proper clinical setting CT: computed tomography

A CT scan showed colonic thickening and induration involving the rectum and sigmoid colon, marked dilatation of the cecum, and severe bilateral centrilobular emphysematous changes in the lungs (Figures 2A, 2B).

The patient was admitted overnight for observation of suspected colitis, leukocytosis, and constipation. The medical team monitored her WBC trend, temperature, and COPD exacerbation and consulted gastroenterology. They prescribed lactulose and Senokot to alleviate her constipation. Over the next two days, the patient experienced abdominal tenderness, constipation, bloating, nausea, bilious vomiting, shortness of breath, wheezing, labored breathing, decreased appetite, and lethargy.

**Figure 3:**
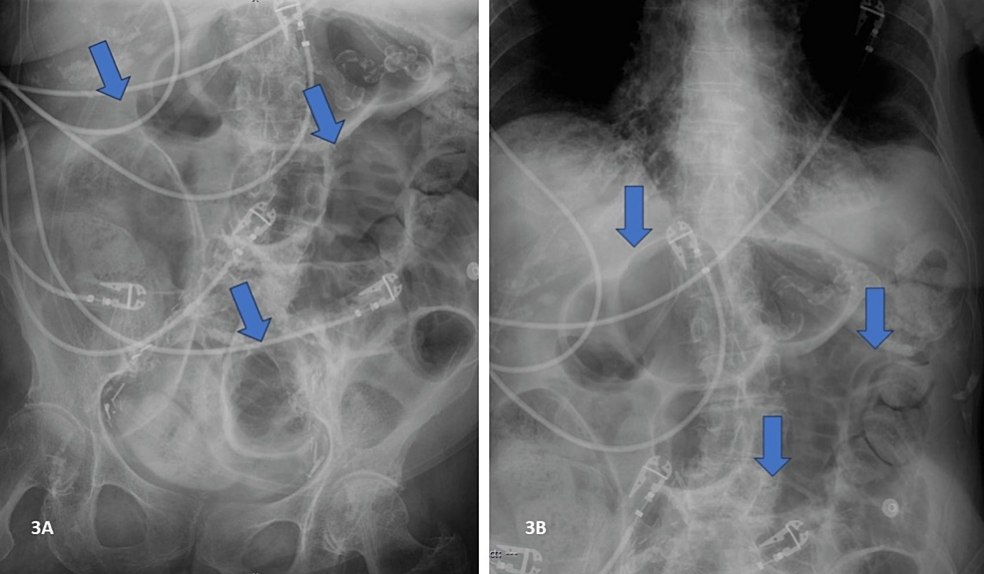
Abdominal radiograph Impression: nonspecific bowel gas pattern. No evidence of intestinal obstruction. No pneumoperitoneum

Lactulose became a concern due to increased bloating, leading to a series of fleet enemas to treat her severe constipation. Zofran helped with the patient's nausea. Her labs one day into observation showed mild hypokalemia, leading to correction with a 20 mEq KCl IV x 1, 40 mEq KCl PO x 1, and D5 NS with 20 mEQ KCl at 75 cc/hr. The use of Solu-Medrol took over the prednisone taper to address her wheezing and marked dyspnea at rest. In addition, an anteroposterior chest X-ray was performed, which showed no evidence of intestinal obstruction. Furthermore, there was a nonspecific bowel gas pattern and no pneumoperitoneum (Figures 3A, 3B).

On the third day of admission, a nasogastric tube was inserted to aid with medication and suction of gastric fluid. The patient had a small bowel movement after seven days of severe constipation. There was minimal improvement in her abdominal distention. During the day, she became increasingly short of breath and had diffuse wheezing. Additionally, the patient became tachycardic, with rates oscillating between 110s and 120s beats per minute. A stat electrocardiogram confirmed sinus tachycardia, while the portable CXR showed no change from prior imaging. A 40 mg IV methylprednisolone was started due to bibasilar atelectasis.

**Figure 4:**
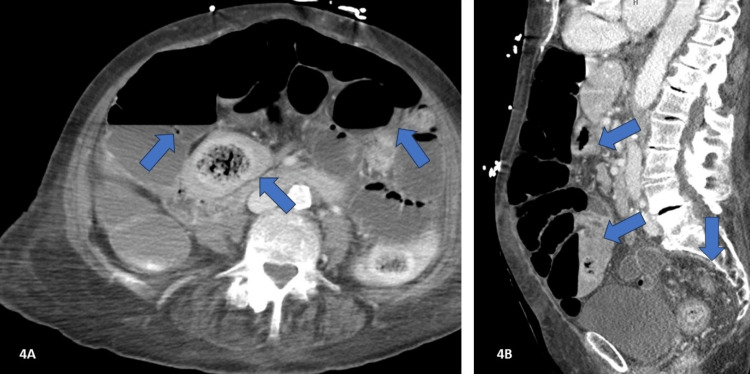
CT with contrast of the abdomen and pelvis - image 2 Impression: a large amount of stool within the colon. There is thickening and induration of the colon, compatible with colitis in the proper clinical setting. There is severe upstream dilatation of the colon and small bowel likely due to obstruction secondary to inflammation and constipation. Recommend surgical consultation. The findings are worse compared to the prior study CT: computed tomography

The patient's condition worsened on the fourth day in the hospital. Despite treatment, her oxygen saturation decreased to 85% due to respiratory distress and agonal breathing. A physical exam revealed severe constipation, and imaging showed obstruction and inflammation (Figures 4A, 4B), prompting a surgical consult.

The patient was recommended to be transferred to the ICU for closer monitoring and treatment. The patient's symptoms progressed during her stay in the ICU, leading to a revised diagnosis of ACPO syndrome or Ogilvie's syndrome. The patient received various treatments, including neostigmine, but surgery was not considered a viable option. Ultimately, the family decided to pursue comfort care, and the patient passed away after being transferred to hospice.

## Discussion

ACPO is a diagnosis of exclusion as its symptoms mimic many other etiologies. ACPO has an estimated incidence rate of 100 cases per 100,000 hospital admissions and a mortality rate of 8%. Of these cases, 15% experience colonic ischemia or perforation, with 40% in this group having severely adverse outcomes [9]. The most common patient profile of Ogilvie’s syndrome is primarily males with an average age of 59.9 years and females with an average age of 56.5 years. Younger females seem to have a higher incidence of ACPO due to higher rates of abdominal procedures among them, such as cesarean sections. This syndrome is not spontaneous, as 94.5% of cases are seen in patients with a medical or surgical condition [8]. Its pathophysiology is not well established, and many comorbid conditions complicate its diagnosis. Our patient’s symptoms of generalized abdominal pain, abdominal tenderness to palpation, constipation lasting several days, nausea, and vomiting initially led to a working differential diagnosis of colitis, leukocytosis, and obstipation. However, her having a single incidence of soft bowel movement complicated the diagnosis. It is reported that 40-50% of patients with ACPO are known to pass flatus [8]. After further radiological testing and failed therapeutics, Ogilvie’s syndrome was included in the working differential diagnosis.

Generally, the following four pathways to acute colonic pseudo-obstruction have been evaluated: chronic disease (primarily cardiovascular, neurological, and respiratory), severe systemic illness, operative insult, and medications (opiates, anticholinergics, and antipsychotics) [1,9]. Chronic disease is thought to potentiate the interstitial cells of Cajal and enteric nervous system dysfunction, along with causing electrolyte disturbances and autonomic imbalance leading to impaired or altered colonic movement. Likewise, colonic dysfunction from severe systemic illness, operative insult, and medications can be due to a triggered inflammatory response, electrolyte disturbances, increased sympathetic drive, and reduced parasympathetic activity. Once colonic motility is affected, it can oscillate between colo-colonic reflex arcs or sensory and motor neuronal feedback, which means dysregulation of stimulus communication. More importantly, disrupted colonic motility can lead to ACPO or Ogilvie’s syndrome [9]. The potential outcomes of ACPO are dilatation, obstruction, ischemia, and perforation [1,8-9]. Data that analyzed 13 years of diagnosed Ogilvie’s syndrome revealed that the mortality rate decreased from 9.4 to 6.4%, which can be attributed to the overdiagnosis of ACPO. Surprisingly, data before that 13-year window stated that ACPO mortality rates were near 30% [9]. This could allude to the evasiveness of ACPO diagnosis or the hesitancy in diagnosing patients with the condition. Perhaps, care providers are not well-versed in recognizing Ogilvie’s syndrome when encountering a complex patient profile. Moreover, the 13-year data revealed that colonic perforation and ischemia occur in 10-20% of patients, with 45% of them succumbing to the condition [9].

Another data set spanning 10 years and involving 48 patients diagnosed with ACPO highlighted that 27% of these patients were female with an average age of 67 years. Of clinical significance, conservative management is administered more frequently to women than their male counterparts (p<0.008) [1]. Our patient’s delayed diagnosis and use of conservative management exceeded the suggested 48-72-hour window. As her condition did not improve on complete bowel rest, NG tube decompression, suppositories and softeners, and management of her comorbidities, early introduction of neostigmine and surgical intervention may have optimized her care. 

Of note, eight out of 11 patients who received neostigmine experienced a resolution of the syndrome with no recurrences. Of those treated, 0-33% experienced cholinergic symptoms after treatment ended. Colonoscopic decompression of the colon led to an improvement of cecal diameter in 73-100% of cases but recurrence of dilatation was seen in 10-65% of cases. When colonoscopic decompression was repeated, 56-87% of patients experienced resolution of ACPO but had a higher risk of future cecal distension. In a study involving 67 Ogilvie’s syndrome patients, 26 had a cecostomy with five dying due to non-procedural reasons, one needing a resection, and 13 experiencing complications [8]. In a larger study that evaluated 106,784 cases of ACPO, 90.5% were medically managed, 2.7% received endoscopy only, 6.3% underwent surgery, and 0.5% had both surgery and colonoscopy. This data revealed that the odds of death were significant in those treated with endoscopy only (p<0.0125). As expected, when the therapy becomes gradually invasive, the morbidity and mortality risks increase [7]. It is important to consider ACPO when patients present with seemingly benign symptoms, like severe constipation, but have many comorbid conditions and are on several prescribed medications. Utilizing the clinical profile and diagnostic modalities available can help form a differential diagnosis that considers uncommon syndromes. Based on this comprehensive assessment, timely care can be provided within the suggested window of therapy.

## Conclusions

A high index of suspicion for ACPO may help in reducing associated morbidity in severely ill or postoperative patients. This case report highlights the importance of monitoring for a constellation of benign symptoms that may worsen the condition of a patient with multiple comorbidities. ACPO is caused by damage to the parasympathetic innervation of the large intestine, which causes its functional obstruction and dilatation. It causes abdominal distension affecting the cecum and right colon and can also involve the rectum. Many acute and chronic illnesses involving the heart, lungs, central and peripheral nervous system, childbirth, infections, trauma, surgery, and medications that interfere with parasympathetic outflow, can cause ACPO. Neostigmine is a widely used anticholinesterase in ACPO patients. It is very effective in the quick reversal of ACPO. However, its use is restricted in the setting of recent myocardial infarction, acidosis, asthma, bradycardia, peptic ulcer disease, and beta-blocker treatment. Bowel rest and abdominal decompression are the mainstays of the treatment, and more aggressive measures like colonoscopy and surgery are used if the patients do not respond to neostigmine. Timely and definitive care, especially for seriously ill or vulnerable patients, can reduce the increased risk of morbidity and mortality rates of this opportunistic syndrome.
